# Prognostic significance of p53 immunoexpression in the survival of oral squamous cell carcinoma patients treated with surgery and neoadjuvant chemotherapy

**DOI:** 10.3892/ol.2013.1627

**Published:** 2013-10-15

**Authors:** LI LI, MANABU FUKUMOTO, DUO LIU

**Affiliations:** 1 Guangdong Key Laboratory for Research and Development of Natural Drugs, Guangdong Medical College, Zhanjiang, Guangdong, P.R. China; 2 Department of Pathology, Institute of Development, Aging and Cancer, Tohoku University, Sendai, Miyagi, Japan; 3 Department of Microsurgery, Dalian University Affiliated Hospital, Dalian, Liaoning, P.R. China

**Keywords:** p53 immunohistochemical expression, prognosis, oral squamous cell carcinoma

## Abstract

p53 status is a key biomarker for a variety of cancer types. However, it remains controversial whether p53 is an effective biomarker in oral squamous cell carcinoma (OSCC), particularly with regard to its prognostic value for OSCC patients with combinational treatment. The aim of the current study was to evaluate the prognostic potential of p53 immunoexpression in samples from OSCC patients treated with surgery only or surgery and neoadjuvant chemotherapy. p53 expression was assessed immunohistochemically in biopsy tissues from 44 OSCC patients with a mean follow-up of 35.6 months. Correlations between p53 status, tumor size (T-classification), lymph node status (N-classification) and clinical outcome were analyzed. It was observed that p53-positive and N0 cases correlated with higher 5-year survival rates in cases treated with surgery alone (P=0.017 and P=0.03, respectively), while in cases with neoadjuvant chemotherapy, p53 status and lymph node status did not exhibit prognostic significance. Tumor size showed no prognostic value in cases receiving surgery alone or in those with neoadjuvant chemotherapy. The present results demonstrated for the first time that p53 immunohistochemical expression correlates with a good prognosis in OSCC patients receiving surgery alone. In conclusion, p53 immunohistochemical expression and lymph node status may serve as prognostic markers for the survival of OSCC patients receiving surgery only, but not for patients undergoing surgery and neoadjuvant chemotherapy treatment.

## Introduction

Oral squamous cell carcinoma (OSCC) is the most frequently occurring malignant tumor of the oral cavity worldwide (1) and continues to be a serious public health issue. In spite of significant advances in therapeutics and early diagnosis, the prognosis of patients with OSCC remains poor (1–3). OSCC tumorigenesis is a complex and multistep process determined by multiple genetic factors (4–8), and the current tumor-node-metastasis (TNM: T, tumor size; N, lymph node status; M, metastasis) classification and histopathological factors, including tumor depth and grade, do not fully predict the clinical outcome in all cases (9). Thus, it is crucial to search for potential molecular markers underlying the development of OSCC and the ability to predict its prognosis, which may be clinically useful for guiding personalized therapy for patients.

The *p53* tumor suppressor gene, located on chromosome 17p13.1, encodes a critical stress response protein that functions primarily as a transcription factor, regulating a large number of genes in response to a variety of cellular insults, including oncogene activation and DNA damage (10). The p53 protein suppresses cellular transformation by inducing growth arrest, apoptosis, DNA repair and differentiation in damaged cells (11). Mutations and alterations in the *p53* gene have been implicated in almost all human cancers, and p53 status is, therefore, one of the most important biomarkers for a variety of cancer types (12,13). Wild-type p53 protein (encoded by the wild-type *p53* gene) has an extremely short half-life and is usually undetectable by immunohistochemistry (IHC) (14). By contrast, mutant p53 protein (encoded by the mutant *p53* gene) is often stabilized by mutations, accumulated at extremely high levels and detectable in tumors by IHC (14). Thus, the immunohistochemical expression of p53 is often used to identify *p53* gene status. At present, the immunohistochemical expression of p53 and its effect on prognosis in OSCC has been investigated in a number of previously published studies, but the data show conflicting results (15–18). Furthermore, the prognostic value of p53 status may vary among patients with different treatment plans.

In the present study, p53 immunohistochemical staining was performed using pre-operative biopsy samples from 44 OSCC patients, and prognostic value was evaluated together with other factors, including lymph node metastasis and tumor size.

## Materials and methods

### Patients and tumor samples

Archival pathological specimens for immunohistochemical study were obtained from the Fuchu Metropolitan Hospital (Tokyo, Japan) and Sendai National Hospital (Sendai, Japan). The specimens consisted of 44 cases of OSCC. The present study was approved by the Ethics Committee of these two hospitals and the Graduate School of Medicine, Tohoku University (Sendai, Miyagi, Japan). The experiments were undertaken with the informed written consent of each patient and the study conformed to the Code of Ethics of the World Medical Association (Declaration of Helsinki). Diagnostic verification and tumor subtyping and grading were independently performed by two certified pathologists. Patients with distant metastases at diagnosis were excluded from this study. T- and N-classification was assigned according to the staging of the Union for International Cancer Control (19). All patients had undergone surgical resection of the tumors and were classified into two groups consisting of 24 patients who had been treated with neoadjuvant chemotherapy and 20 who had been treated with surgery alone. Chemotherapy consisted of ~70 mg/m^2^ cisplatinum and 550 mg/m^2^ 5-fluorouracil. Surgery was performed four to six weeks after neoadjuvant therapy. The clinicopathological characteristics of the patients are shown in Table I. The mean follow-up period was 35.6 months (range, 5–95.3 months).

### IHC

Histological specimens for diagnosis that were obtained from a diagnostic biopsy underwent standard immunohistochemical staining for the p53 protein. Briefly, formalin-fixed, paraffin-embedded archived tissue blocks were sectioned at 4 μm and transferred to microscope slides. The sections were deparaffinized in xylene and rehydrated in ethanol solution. Antigen retrieval was performed in 10 mM citrate buffer (pH 6.0) using a microwave (15 min; 100°C) and cooled to room temperature. Endogenous peroxidase was blocked with 0.3% H_2_O_2_ in methanol for 30 min. Non-specific binding was blocked with 2.5% skimmed milk for 20 min at room temperature. Following rinsing with wash buffer, sections were incubated overnight at 4°C with anti-human p53 mouse monoclonal antibody (Clone DO-7; Dako, Carpinteria, CA, USA) at a 1:400 dilution. Subsequently, biotinylated goat anti-mouse antibody and an ABC kit (both Dako) were used for detection. The sections were developed with diaminobenzidine tetrahydrochloride (Dojin, Kumamoto, Japan) and counterstained with hematoxylin. Negative controls were employed in which the primary antibody was replaced by phosphate-buffered saline. Positively stained cells were counted under a microscope using ×200 magnification in a minimum of five selected areas with frequent positive staining. A minimum of 2,000 cells were counted in each section. The tumor was considered p53-positive if ≥10% of the nuclei of the tumor cells were positively stained.

### Statistical analysis

For the statistical analyses, SPSS software (SPPS for Windows, version 12.0; SPSS, Inc., Chicago, IL, USA) was utilized. The Kaplan-Meier method was used to assess actual 5-year survival rates and the differences between groups were analyzed by a log-rank test. For all analyses, P<0.05 was considered to indicate a statistically significant difference.

## Results

### Expression of p53 protein in OSCC

**Figure 1:**
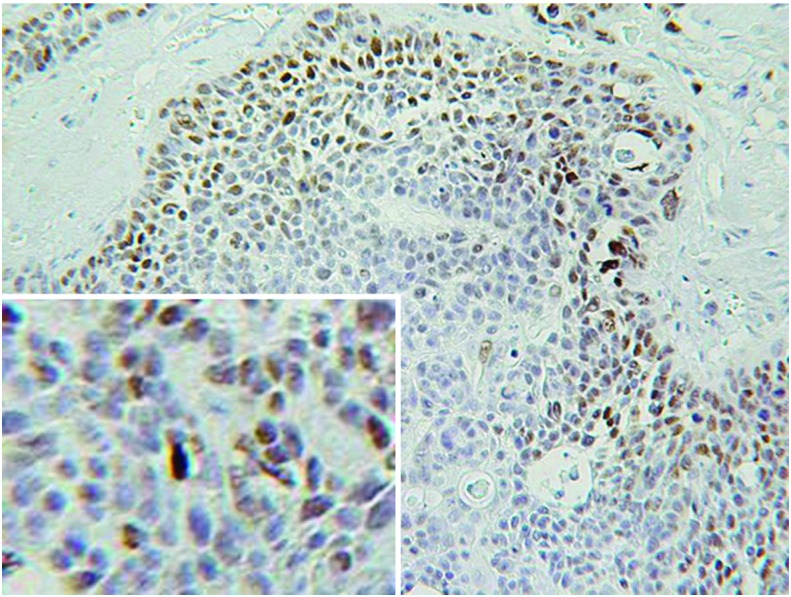
Representative immunohistochemical staining for p53 protein in OSCC. Original magnification, ×200; brown nuclear staining with diaminobenzidine tetrahydrochloride. OSCC, oral squamous cell carcinoma.

Immunohistochemical staining showed that 21 of the 44 specimens (47.7%) examined were p53-positive. p53 protein was exclusively expressed in the nuclei and not in the cytoplasm of the cancer cells (Fig. 1). p53 was mainly expressed in the invasive front of the cancer cell nest.

### p53 expression in OSCC and its prognostic significance

**Figure 2:**
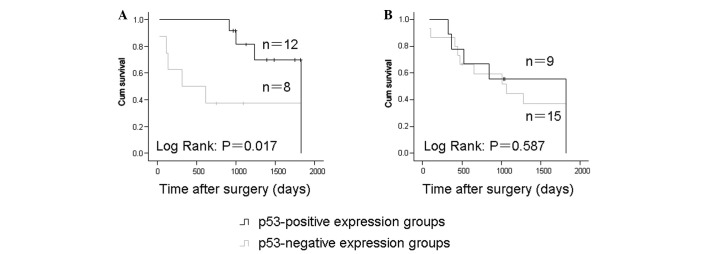
Kaplan-Meier five-year survival curves showing the association between p53 expression and the survival of 44 OSCC patients. (A) Cases with p53-positive expression showed significantly favorable prognoses where patients had received surgery alone. (B) No significant differentiation in cumulative survival time was observed in cases where patients had received neoadjuvant chemotherapy. OSCC, oral squamous cell carcinoma.

Among the cases treated with surgery alone, the five-year survival rates were 25 and 58.3% for the p53-negative and -positive expression groups, respectively. In addition, the p53-positive expression group showed a significantly higher survival rate compared with the p53-negative expression group (P=0.017; Fig. 2A). No significant correlation between p53 expression and patient survival was observed in the neoadjuvant chemotherapy group (P=0.385; Fig. 2B).

### N-classification and prognosis

**Figure 3:**
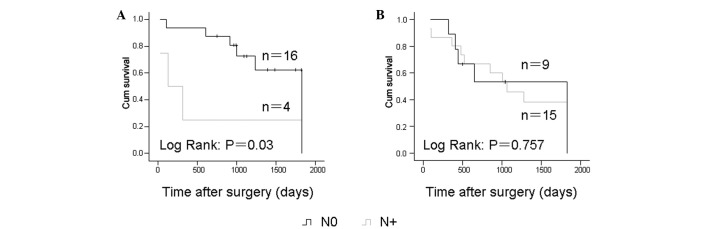
Kaplan-Meier curves of five-year survival times of OSCC patients according to the lymph node status. Cases who received (A) surgery alone and (B) neoadjuvant chemotherapy. OSCC, oral squamous cell carcinoma; N, lymph node status.

Analysis of Kaplan-Meier survival curves showed that OSCC patients with positive lymph node metastasis had significantly shorter overall survival times compared with others among the cases receiving surgery alone (P=0.03). Similar to the p53 expression status, the N status was not significantly correlated with survival in the chemotherapy group (Fig. 3).

### T-classification and prognosis

**Figure 4:**
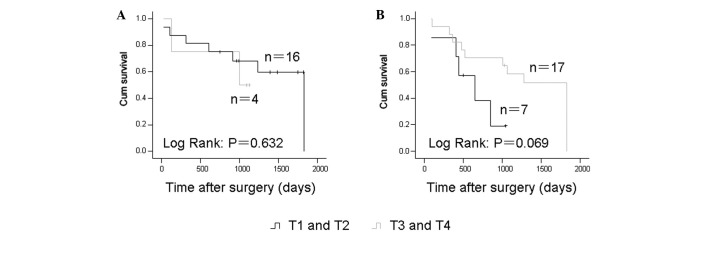
Kaplan-Meier curves of five-year survival times of OSCC patients according to the tumor size. Cases who received (A) surgery alone and (B) neoadjuvant chemotherapy. OSCC, oral squamous cell carcinoma; T, tumor size.

The T-classification and prognosis were further evaluated in these samples. In the cases receiving surgery alone and in the cases who underwent neoadjuvant chemotherapy, the T-classification showed no correlation with the five-year survival rate (Fig. 4).

## Discussion

In the present study, biopsy samples were selected instead of surgical resection samples for examination. This was as clinical doctors are able to obtain biopsy samples earlier than surgical resection samples and an early prognostic evaluation is useful for guiding treatment. However, p53 expression showed no significant difference between the biopsy and surgical resection samples in the present study (data not shown).

TNM staging is used as a standard system for the prediction of the prognosis of OSCC, however, certain studies have shown that even among patients of the same stage, patient prognoses are discordant (20). In the present study, superior patient survival was observed with surgery alone with N0 compared with N^+^, which is consistent with previous studies (21,22). Nevertheless, certain studies have reported that the N-classification does not correlate with the survival of the patients (23,24). No significant correlation was identified between the T-classification and patient survival. Similar to the N-classification, the correlation between the T-classification and survival remains controversial (3,9). Such conflicting results may be due to the fact that the TNM system only considers the anatomical characteristics of the tumors without considering the biological and molecular characteristics.

In the present study, the level of positive p53 expression in the OSCC patient samples was 47.7%, a similar level to that observed in previous studies (18,25). Additionally, the present study showed that p53 was often observed to be expressed in the outer layer of the cancer cell nest, the location of the aggressive tumor invasion, which is consistent with the results of a previous study (17). Contrary to our predictions, the results of the present study showed that p53-positive expression in the biopsy of OSCC patients undergoing surgery only is an indicator of an improved survival time. To the best of our knowledge, only two types of results have been reported concerning the association between the immunohistochemical expression of p53 and the prognosis of OSCC, namely, the negative expression of p53 indicating an improved prognosis or no correlation between p53 expression and prognosis (15–18). p53 mutation results in not only the loss of tumor suppressor function, but also in the gain of new oncogenic properties, including increasing the tumor formation ability and drug resistance (13). The results of the present study, therefore, do not appear to be consistent with the concept of p53 mutation. There are several possible reasons for this inconsistency.

Firstly, p53 immunohistochemical staining does not always indicate the p53 mutation status. Certain p53 mutations are IHC-null/negative (14), while specific tumors under continuous stress stimulation lead to accumulation of functional wild-type p53 protein (26). Secondly, studies of tumor cell lines and mouse tumor models have shown that oncogene activation and abnormal proliferation are able to trigger apoptosis through the coupling of the signal transduction pathway of apoptosis and cell proliferation through p53-dependent and -independent mechanisms, and the p53 mutation may lead to higher cell apoptosis levels (27,28). When these apoptosis levels reach a certain threshold, it may affect survival. As an example, Zheng *et al*(29) reported that intestinal-type gastric carcinomas with a more favorable prognosis frequently exhibited elevated levels of proliferation and apoptosis accompanied by a higher expression level of mutant p53 compared with diffuse-type carcinomas, with a higher degree of malignancy. Notably, one recent study reported that mutant p53 dictated an improved chemotherapy response in a mouse mammary gland tumor model compared with wild-type p53 due to the mechanism whereby wild-type p53-mediated senescence impairs the apoptotic response to chemotherapy and the clinical outcome in breast cancer (30). Therefore, p53 status may be a good factor for the evaluation of OSCC prognosis when considered together with other factors, such as apoptosis and the senescence level. In addition, in the present study, p53-positive expression and N0 were statistically correlated with a good prognosis in OSCC patients receiving surgery alone, but not in the chemotherapy group, which may be due to the fact that chemotherapy completely disrupted the inherent apoptosis mechanisms of the tumor cells.

In summary, the results of the present study showed that p53-positive immunoexpression and N0 status indicates an improved prognosis in OSCC patients receiving surgery alone, but not in patients undergoing surgery and neoadjuvant chemotherapy. p53 status may serve as a good prognostic factor for the survival of OSCC patients when combined with other factors, such as apoptosis and senescence. Considering the inherent limitations of IHC studies, it may be necessary to combine the IHC study with *p53* gene exon sequencing to confirm the p53 mutation status in future studies.

## Figures and Tables

**Table I:** Clinicopathological characteristics of 44 OSCC patients.

Parameters	Patients, n
Gender	
Male	28
Female	16
Age, years	
37–83 (64.6±11.1)^a^	44
Primary lesion	
Tongue	22
Lower gingiva	10
Floor of the mouth	5
Buccal mucosa	3
Upper gingiva	4
T	
T1	5
T2	18
T3	8
T4	13
N	
N0	25
N^+^	19
Neoadjuvant treatment	
Chemotherapy	24
None	20
Total	44

a Age rage (mean ± standard deviation).

OSCC, oral squamous cell carcinoma; T, tumor size; N, lymph node status.
